# *trans*-Diaqua­bis­[2,5-bis­(pyridin-2-yl)-1,3,4-thia­diazole]cobalt(II) bis­(tetra­fluoridoborate)

**DOI:** 10.1107/S1600536811020125

**Published:** 2011-06-04

**Authors:** Fouad Bentiss, Frédéric Capet, Michel Lagrenée, Mohamed Saadi, Lahcen El Ammari

**Affiliations:** a Laboratoire de Chimie de Coordination et d’Analytique (LCCA), Faculté des Sciences, Université Chouaib Doukkali, BP 20, M-24000 El Jadida, Morocco; b Unité de Catalyse et de Chimie du Solide (UCCS), CNRS UMR 8181, ENSCL, BP 90108, F-59652 Villeneuve d’Ascq Cedex, France; c Université Lille Nord de France, F-59000 Lille, France; d Laboratoire de Chimie du Solide Appliquée, Faculté des Sciences, Université Mohammed V-Agdal, Avenue Ibn Battouta, BP 1014, Rabat, Morocco

## Abstract

The bidentate 1,3,4-thia­diazole ligand substituted by two 2-pyridyl rings (denoted *L*) has been found to produce the new monomeric title complex, [Co(C_12_H_8_N_4_S)_2_(H_2_O)_2_](BF_4_)_2_. The thia­diazole and pyridyl rings surrounding the Co atom are almost coplanar [dihedral angle = 4.35 (7)°]. The mean plane defined by these heterocyclic moieties makes a dihedral angle of 18.72 (6)° with the non-coordinated pyridyl ring. The Co^2+^ cation, located at a crystallographic center of symmetry, is bonded to two ligands and two water mol­ecules in a *trans* configuration in an octa­hedral environment. The tetra­fluorido­­borate ions can be regarded as free anions in the crystal lattice. Nevertheless, they are involved in an infinite two-dimensional network along  the [010] and [101] directions of O—H⋯F hydrogen bonds.

## Related literature

For background to compounds with the same ligand, see: Bentiss *et al.* (2002 ▶, 2004 ▶); Zheng *et al.* (2006 ▶). For an improved synthesis of the ligand, see: Lebrini *et al.* (2005 ▶).
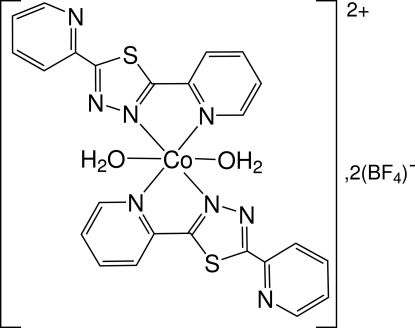


## Experimental

### 

#### Crystal data

[Co(C_12_H_8_N_4_S)_2_(H_2_O)_2_](BF_4_)_2_
*M* *_r_* = 749.15
Monoclinic, 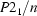

*a* = 10.8319 (2) Å
*b* = 11.0623 (2) Å
*c* = 13.2120 (3) Å
β = 101.114 (1)°
*V* = 1553.45 (5) Å^3^
*Z* = 2
Mo *K*α radiation
μ = 0.77 mm^−1^
*T* = 100 K
0.39 × 0.30 × 0.19 mm

#### Data collection

Bruker X8 APEXII diffractometer
Absorption correction: multi-scan (*SADABS*; Bruker, 2005 ▶) *T*_min_ = 0.757, *T*_max_ = 0.863
32328 measured reflections
3055 independent reflections
2877 reflections with *I* > 2σ(*I*)
*R*_int_ = 0.023

#### Refinement

*R*[*F* ^2^ > 2σ(*F* ^2^)] = 0.025
*wR*(*F* ^2^) = 0.064
*S* = 1.04
3055 reflections
214 parameters
H-atom parameters constrained
Δρ_max_ = 0.54 e Å^−3^
Δρ_min_ = −0.36 e Å^−3^

### 

Data collection: *APEX2* (Bruker, 2005 ▶); cell refinement: *SAINT* (Bruker, 2005 ▶); data reduction: *SAINT*; program(s) used to solve structure: *SHELXS97* (Sheldrick, 2008 ▶); program(s) used to refine structure: *SHELXL97* (Sheldrick, 2008 ▶); molecular graphics: *ORTEP-3 for Windows* (Farrugia, 1997 ▶); software used to prepare material for publication: *WinGX* (Farrugia, 1999 ▶).

## Supplementary Material

Crystal structure: contains datablock(s) I, global. DOI: 10.1107/S1600536811020125/im2290sup1.cif

Structure factors: contains datablock(s) I. DOI: 10.1107/S1600536811020125/im2290Isup2.hkl

Additional supplementary materials:  crystallographic information; 3D view; checkCIF report

## Figures and Tables

**Table 1:** Hydrogen-bond geometry (Å, °)

*D*—H⋯*A*	*D*—H	H⋯*A*	*D*⋯*A*	*D*—H⋯*A*
O1—H1*W*⋯F1^i^	0.86	1.88	2.7014 (16)	161
O1—H2*W*⋯F4	0.86	1.94	2.7927 (16)	172

Symmetry code: (i) 
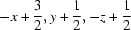
.

**Fig. 1.**
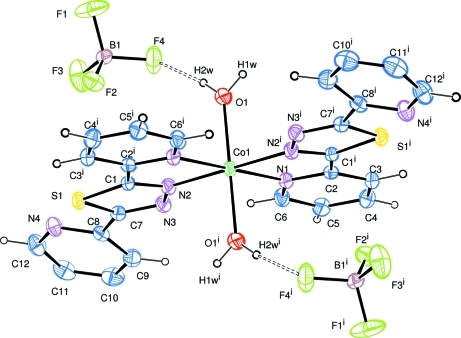
Molecular structure showing the cationic cobalt complex and the counter ions. Displacement ellipsoids are drawn at the 50% probability level. H atoms are represented as small circles. Hydrogen bonds are depicted as dashed lines.

## supplementary crystallographic information

### Comment

2,5-bis(2-pyridyl)-1,3,4-thiadiazole can be used to produce molecular architectures with transition metals in association with anionic co-ligands. In the resulting di- and mononuclear complexes, a variety of coordination modes have been observed, of which the dinuclear (N`N``, N2, N``) bridging, the dinuclear (N`N``, N2, N``)2 double bridging and the monoclear (N`,*N*`)2 coordination mode are the most common and the most important ones (Scheme 1). In the latter case, the *trans*-configuration is exclusively observed for octahedral complexes.

The structures of monomeric complexes of the neutral 2,5-bis(2-pyridyl)-1,3, 4-thiadiazole derivative with divalent Zn (tetrachloride and perchlorate), Co (nitrate and perchlorate), Ni (perchlorate) and Cu (nitrate and perchlorate) have been previously reported (Bentiss *et al.*,2002, 2004; Zheng *et al.* 2006). We report here the synthesis and the single-crystal structure of the new monomeric cobalt complex formed by 2,5-bis(2-pyridyl)-1,3, 4-thiadiazole with tetrafluoroborates as counter ions.

The complex cation shows an almost regular octahedral environment of cobalt cation which is located at a crystallographic center of symmetry. Cobalt therefore is linked to two ligands and two water molecules as shown in Fig.1. As a matter of fact, the cobalt coordination sphere is achieved by interaction with the nitrogen atom of a single pyridyl ring and with the adjacent nitrogen of the azine group with Co—N distances in the range of 2.082 (2)— 2.173 (2) Å. Moreover, the water molecules are found in axial positions at distances of Co—O 2.128 (2) Å and all N—Co—O angles being close to 90 °.

The dihedral angle between thiadiazole and the coordinating pyridyl ring is 4.35 (7)°. The mean plane defined by the two preceding heterocyclic moieties forms a dihedral angle with the non-coordinating pyridine ring (N4-C8-C9-C10-C11-C12) of 18.72 (6)°. The counter ion, BF_4_^-^, is involved in an infinite two-dimensional network of hydrogen bonds (Table 1).

### Experimental

2,5-Bis(2-pyridyl)-1,3,4-thiadiazole ligand (noted *L*) was synthesized as described previously by Lebrini *et al.*, 2005. Co(BF_4_)_2_ × 6 H_2_O (1.5 mmol, 0.51 g) in 8 ml of water was added to (0.42 mmol, 0.1 g) of *L* (bptd ligand) dissolved in 8 ml of ethanol. The solution was filtered and after 24 h, the orange compound crystallized at room temperature. Crystals were washed with water and dried under vacuum. Yield: 54%. Anal. Calc. for C_12_H_10_BCo_0.5_F_4_N_4_OS: C, 38.44; H, 2.67; N, 14.95; S, 8.56; F, 20.29%. Found: C, 38.56; H, 2.72; N, 14.88; S, 8.51; F, 20.36%.

### Refinement

H atoms were located in a difference map and treated as riding with C—H = 0.95 Å for the aromatic CH, with *U*_iso_(H) = 1.2 *U*_eq_ (aromatic). The O-bound H atom is initially located in a difference map and refined with O—H distance restraints of 0.86 (1). In a the last cycle there is refined in the riding model approximation with *U*_iso_(H) set to 1.2*U*_eq_(O).

### Crystal data

**Table:**

[Co(C_12_H_8_N_4_S)_2_(H_2_O)_2_](BF_4_)_2_	*F*(000) = 754
*M**_r_* = 749.15	*D*_x_ = 1.602 Mg m^−^^3^
Monoclinic, *P*2_1_/*n*	Mo *K*α radiation, λ = 0.71073 Å
Hall symbol: -P 2yn	Cell parameters from 3055 reflections
*a* = 10.8319 (2) Å	θ = 2.5–26.0°
*b* = 11.0623 (2) Å	µ = 0.77 mm^−^^1^
*c* = 13.2120 (3) Å	*T* = 100 K
β = 101.114 (1)°	Prism, pink
*V* = 1553.45 (5) Å^3^	0.39 × 0.30 × 0.19 mm
*Z* = 2	

### Data collection

**Table:**

Bruker X8 APEXII diffractometer	3055 independent reflections
Radiation source: fine-focus sealed tube	2877 reflections with *I* > 2σ(*I*)
graphite	*R*_int_ = 0.023
φ and ω scans	θ_max_ = 26.0°, θ_min_ = 2.4°
Absorption correction: multi-scan (*SADABS*; Bruker, 2005)	*h* = −13→13
*T*_min_ = 0.757, *T*_max_ = 0.863	*k* = −13→13
32328 measured reflections	*l* = −16→16

### Refinement

**Table:**

Refinement on *F*^2^	Primary atom site location: structure-invariant direct methods
Least-squares matrix: full	Secondary atom site location: difference Fourier map
*R*[*F*^2^ > 2σ(*F*^2^)] = 0.025	Hydrogen site location: inferred from neighbouring sites
*wR*(*F*^2^) = 0.064	H-atom parameters constrained
*S* = 1.04	*w* = 1/[σ^2^(*F*_o_^2^) + (0.028*P*)^2^ + 1.2095*P*] where *P* = (*F*_o_^2^ + 2*F*_c_^2^)/3
3055 reflections	(Δ/σ)_max_ = 0.001
214 parameters	Δρ_max_ = 0.54 e Å^−^^3^
0 restraints	Δρ_min_ = −0.36 e Å^−^^3^

### Special details

**Table:**

Geometry. All e.s.d.'s (except the e.s.d. in the dihedral angle between two l.s. planes) are estimated using the full covariance matrix. The cell e.s.d.'s are taken into account individually in the estimation of e.s.d.'s in distances, angles and torsion angles; correlations between e.s.d.'s in cell parameters are only used when they are defined by crystal symmetry. An approximate (isotropic) treatment of cell e.s.d.'s is used for estimating e.s.d.'s involving l.s. planes.
Refinement. Refinement of *F*^2^ against all reflections. The weighted *R*-factor *wR* and goodness of fit *S* are based on *F*^2^, conventional *R*-factors *R* are based on *F*, with *F* set to zero for negative *F*^2^. The threshold expression of *F*^2^ > σ(*F*^2^) is used only for calculating *R*-factors(gt) *etc*. and is not relevant to the choice of reflections for refinement. *R*-factors based on *F*^2^ are statistically about twice as large as those based on *F*, and *R*- factors based on all data will be even larger.

### Fractional atomic coordinates and isotropic or equivalent isotropic displacement parameters (Å^2^)

**Table:**

	*x*	*y*	*z*	*U*_iso_*/*U*_eq_	
B1	0.57309 (16)	0.64485 (16)	0.25732 (14)	0.0216 (3)	
F1	0.67597 (12)	0.59356 (14)	0.32121 (9)	0.0571 (4)	
F2	0.58207 (9)	0.62506 (10)	0.15136 (7)	0.0332 (2)	
F3	0.46504 (11)	0.59451 (11)	0.27939 (11)	0.0506 (3)	
F4	0.57202 (12)	0.76967 (9)	0.27725 (9)	0.0446 (3)	
C1	0.40758 (13)	0.75359 (13)	−0.02371 (11)	0.0182 (3)	
C2	0.47872 (13)	1.25525 (13)	0.07062 (11)	0.0183 (3)	
C3	0.43647 (14)	1.36225 (14)	0.10807 (12)	0.0217 (3)	
H3	0.4818	1.4354	0.1061	0.026*	
C4	0.32601 (15)	1.36036 (14)	0.14872 (12)	0.0247 (3)	
H4	0.2948	1.4323	0.1740	0.030*	
C5	0.26323 (15)	1.25161 (14)	0.15134 (12)	0.0245 (3)	
H5	0.1882	1.2476	0.1784	0.029*	
C6	0.31236 (14)	1.14767 (14)	0.11331 (12)	0.0224 (3)	
H6	0.2695	1.0732	0.1161	0.027*	
C7	0.22011 (13)	0.74718 (13)	0.04650 (11)	0.0196 (3)	
C8	0.10139 (14)	0.71559 (14)	0.07978 (11)	0.0206 (3)	
C9	0.01592 (15)	0.80486 (16)	0.09485 (13)	0.0278 (3)	
H9	0.0326	0.8880	0.0854	0.033*	
C10	−0.09485 (16)	0.76752 (18)	0.12427 (14)	0.0327 (4)	
H10	−0.1560	0.8253	0.1349	0.039*	
C11	−0.11496 (16)	0.64530 (17)	0.13791 (13)	0.0317 (4)	
H11	−0.1899	0.6180	0.1578	0.038*	
C12	−0.02334 (16)	0.56355 (16)	0.12188 (13)	0.0295 (4)	
H12	−0.0372	0.4800	0.1322	0.035*	
Co1	0.5000	1.0000	0.0000	0.01779 (9)	
N1	0.41821 (12)	1.14819 (11)	0.07273 (10)	0.0196 (3)	
N2	0.37922 (11)	0.85730 (11)	0.01641 (10)	0.0195 (3)	
N3	0.27082 (12)	0.85445 (11)	0.05733 (10)	0.0210 (3)	
N4	0.08401 (12)	0.59657 (12)	0.09253 (10)	0.0243 (3)	
O1	0.61489 (10)	0.95481 (10)	0.14452 (9)	0.0267 (2)	
H1W	0.6833	0.9925	0.1697	0.032*	
H2W	0.6079	0.9000	0.1892	0.032*	
S1	0.30230 (3)	0.64074 (3)	−0.01342 (3)	0.01990 (10)	

### Atomic displacement parameters (Å^2^)

**Table:**

	*U*^11^	*U*^22^	*U*^33^	*U*^12^	*U*^13^	*U*^23^
B1	0.0179 (8)	0.0198 (8)	0.0261 (9)	0.0022 (6)	0.0013 (7)	0.0020 (7)
F1	0.0467 (7)	0.0832 (10)	0.0362 (6)	0.0401 (7)	−0.0049 (5)	0.0013 (6)
F2	0.0322 (5)	0.0381 (6)	0.0277 (5)	−0.0042 (4)	0.0018 (4)	−0.0023 (4)
F3	0.0409 (6)	0.0405 (7)	0.0801 (9)	−0.0129 (5)	0.0361 (6)	−0.0168 (6)
F4	0.0673 (8)	0.0217 (5)	0.0455 (6)	−0.0078 (5)	0.0126 (6)	−0.0022 (5)
C1	0.0168 (7)	0.0148 (7)	0.0213 (7)	−0.0023 (5)	−0.0009 (5)	0.0027 (5)
C2	0.0170 (7)	0.0176 (7)	0.0185 (7)	−0.0009 (5)	−0.0012 (5)	0.0018 (5)
C3	0.0230 (8)	0.0164 (7)	0.0250 (8)	−0.0022 (6)	0.0024 (6)	0.0006 (6)
C4	0.0273 (8)	0.0204 (8)	0.0270 (8)	0.0026 (6)	0.0070 (6)	−0.0017 (6)
C5	0.0224 (7)	0.0252 (8)	0.0268 (8)	−0.0006 (6)	0.0074 (6)	0.0007 (6)
C6	0.0213 (7)	0.0198 (7)	0.0266 (8)	−0.0046 (6)	0.0061 (6)	0.0006 (6)
C7	0.0178 (7)	0.0185 (7)	0.0212 (7)	−0.0003 (6)	0.0006 (6)	0.0010 (6)
C8	0.0177 (7)	0.0232 (8)	0.0201 (7)	−0.0033 (6)	0.0018 (5)	−0.0013 (6)
C9	0.0220 (8)	0.0259 (8)	0.0348 (9)	0.0002 (6)	0.0032 (7)	−0.0001 (7)
C10	0.0219 (8)	0.0428 (10)	0.0341 (9)	0.0051 (7)	0.0074 (7)	−0.0038 (8)
C11	0.0226 (8)	0.0463 (11)	0.0284 (9)	−0.0080 (7)	0.0103 (7)	−0.0017 (8)
C12	0.0305 (9)	0.0300 (9)	0.0302 (8)	−0.0106 (7)	0.0114 (7)	−0.0016 (7)
Co1	0.01571 (14)	0.01254 (14)	0.02481 (16)	−0.00254 (10)	0.00315 (11)	−0.00077 (10)
N1	0.0184 (6)	0.0159 (6)	0.0235 (6)	−0.0018 (5)	0.0017 (5)	0.0012 (5)
N2	0.0166 (6)	0.0164 (6)	0.0253 (6)	−0.0018 (5)	0.0033 (5)	−0.0002 (5)
N3	0.0173 (6)	0.0194 (6)	0.0264 (7)	−0.0028 (5)	0.0043 (5)	0.0004 (5)
N4	0.0239 (7)	0.0229 (7)	0.0275 (7)	−0.0059 (5)	0.0083 (5)	−0.0016 (5)
O1	0.0244 (6)	0.0220 (6)	0.0306 (6)	−0.0050 (4)	−0.0027 (5)	0.0039 (5)
S1	0.01719 (18)	0.01395 (17)	0.0284 (2)	−0.00295 (13)	0.00399 (14)	0.00011 (14)

### Geometric parameters (Å, °)

**Table:**

B1—F3	1.377 (2)	C7—S1	1.7543 (15)
B1—F1	1.383 (2)	C8—N4	1.345 (2)
B1—F4	1.406 (2)	C8—C9	1.394 (2)
B1—F2	1.439 (2)	C9—C10	1.393 (2)
C1—N2	1.3245 (19)	C9—H9	0.9500
C1—C2^i^	1.485 (2)	C10—C11	1.387 (3)
C1—S1	1.7138 (14)	C10—H10	0.9500
C2—N1	1.3565 (19)	C11—C12	1.389 (3)
C2—C3	1.394 (2)	C11—H11	0.9500
C2—C1^i^	1.485 (2)	C12—N4	1.345 (2)
C3—C4	1.403 (2)	C12—H12	0.9500
C3—H3	0.9500	Co1—N2	2.0880 (12)
C4—C5	1.386 (2)	Co1—N2^i^	2.0880 (12)
C4—H4	0.9500	Co1—O1^i^	2.1280 (11)
C5—C6	1.400 (2)	Co1—O1	2.1280 (11)
C5—H5	0.9500	Co1—N1^i^	2.1734 (13)
C6—N1	1.356 (2)	Co1—N1	2.1734 (13)
C6—H6	0.9500	N2—N3	1.3845 (17)
C7—N3	1.3039 (19)	O1—H1W	0.8597
C7—C8	1.479 (2)	O1—H2W	0.8597
			
F3—B1—F1	108.81 (15)	C9—C10—H10	120.3
F3—B1—F4	108.63 (14)	C10—C11—C12	118.70 (15)
F1—B1—F4	108.86 (14)	C10—C11—H11	120.6
F3—B1—F2	111.36 (14)	C12—C11—H11	120.6
F1—B1—F2	109.49 (13)	N4—C12—C11	123.37 (16)
F4—B1—F2	109.65 (13)	N4—C12—H12	118.3
N2—C1—C2^i^	119.94 (13)	C11—C12—H12	118.3
N2—C1—S1	112.95 (11)	N2—Co1—N2^i^	180.0
C2^i^—C1—S1	127.10 (11)	N2—Co1—O1^i^	90.06 (5)
N1—C2—C3	122.76 (13)	N2^i^—Co1—O1^i^	89.94 (5)
N1—C2—C1^i^	113.20 (13)	N2—Co1—O1	89.94 (5)
C3—C2—C1^i^	124.03 (13)	N2^i^—Co1—O1	90.06 (5)
C2—C3—C4	119.07 (14)	O1^i^—Co1—O1	180.0
C2—C3—H3	120.5	N2—Co1—N1^i^	78.03 (5)
C4—C3—H3	120.5	N2^i^—Co1—N1^i^	101.97 (5)
C5—C4—C3	118.77 (14)	O1^i^—Co1—N1^i^	89.90 (5)
C5—C4—H4	120.6	O1—Co1—N1^i^	90.10 (5)
C3—C4—H4	120.6	N2—Co1—N1	101.97 (5)
C4—C5—C6	118.80 (14)	N2^i^—Co1—N1	78.03 (5)
C4—C5—H5	120.6	O1^i^—Co1—N1	90.10 (5)
C6—C5—H5	120.6	O1—Co1—N1	89.90 (5)
N1—C6—C5	123.17 (14)	N1^i^—Co1—N1	180.0
N1—C6—H6	118.4	C6—N1—C2	117.42 (13)
C5—C6—H6	118.4	C6—N1—Co1	128.27 (10)
N3—C7—C8	123.68 (14)	C2—N1—Co1	114.19 (10)
N3—C7—S1	114.88 (11)	C1—N2—N3	114.59 (12)
C8—C7—S1	121.44 (11)	C1—N2—Co1	114.52 (10)
N4—C8—C9	124.24 (14)	N3—N2—Co1	130.79 (9)
N4—C8—C7	114.78 (13)	C7—N3—N2	110.42 (12)
C9—C8—C7	120.97 (14)	C8—N4—C12	116.84 (14)
C10—C9—C8	117.47 (16)	Co1—O1—H1W	123.1
C10—C9—H9	121.3	Co1—O1—H2W	131.9
C8—C9—H9	121.3	H1W—O1—H2W	105.0
C11—C10—C9	119.38 (16)	C1—S1—C7	87.15 (7)
C11—C10—H10	120.3		
			
N1—C2—C3—C4	−0.8 (2)	O1—Co1—N1—C2	91.44 (10)
C1^i^—C2—C3—C4	178.56 (14)	N1^i^—Co1—N1—C2	7.1 (4)
C2—C3—C4—C5	0.7 (2)	C2^i^—C1—N2—N3	179.69 (12)
C3—C4—C5—C6	0.0 (2)	S1—C1—N2—N3	−0.59 (16)
C4—C5—C6—N1	−0.7 (2)	C2^i^—C1—N2—Co1	2.91 (17)
N3—C7—C8—N4	−160.18 (14)	S1—C1—N2—Co1	−177.38 (6)
S1—C7—C8—N4	20.91 (18)	N2^i^—Co1—N2—C1	−137 (5)
N3—C7—C8—C9	20.5 (2)	O1^i^—Co1—N2—C1	89.03 (11)
S1—C7—C8—C9	−158.44 (12)	O1—Co1—N2—C1	−90.97 (11)
N4—C8—C9—C10	−0.5 (2)	N1^i^—Co1—N2—C1	−0.85 (10)
C7—C8—C9—C10	178.78 (14)	N1—Co1—N2—C1	179.15 (10)
C8—C9—C10—C11	0.5 (2)	N2^i^—Co1—N2—N3	47 (5)
C9—C10—C11—C12	0.1 (3)	O1^i^—Co1—N2—N3	−87.11 (12)
C10—C11—C12—N4	−0.8 (3)	O1—Co1—N2—N3	92.89 (12)
C5—C6—N1—C2	0.7 (2)	N1^i^—Co1—N2—N3	−176.99 (13)
C5—C6—N1—Co1	−174.93 (11)	N1—Co1—N2—N3	3.01 (13)
C3—C2—N1—C6	0.0 (2)	C8—C7—N3—N2	−178.29 (13)
C1^i^—C2—N1—C6	−179.34 (12)	S1—C7—N3—N2	0.69 (16)
C3—C2—N1—Co1	176.30 (11)	C1—N2—N3—C7	−0.07 (18)
C1^i^—C2—N1—Co1	−3.08 (15)	Co1—N2—N3—C7	176.07 (10)
N2—Co1—N1—C6	−2.87 (14)	C9—C8—N4—C12	−0.1 (2)
N2^i^—Co1—N1—C6	177.13 (14)	C7—C8—N4—C12	−179.41 (14)
O1^i^—Co1—N1—C6	87.21 (13)	C11—C12—N4—C8	0.7 (2)
O1—Co1—N1—C6	−92.79 (13)	N2—C1—S1—C7	0.78 (11)
N1^i^—Co1—N1—C6	−177.1 (5)	C2^i^—C1—S1—C7	−179.52 (13)
N2—Co1—N1—C2	−178.64 (10)	N3—C7—S1—C1	−0.86 (12)
N2^i^—Co1—N1—C2	1.36 (10)	C8—C7—S1—C1	178.15 (13)
O1^i^—Co1—N1—C2	−88.56 (10)		

Symmetry codes: (i) −*x*+1, −*y*+2, −*z*.

### Hydrogen-bond geometry (Å, °)

**Table:**

*D*—H···*A*	*D*—H	H···*A*	*D*···*A*	*D*—H···*A*
O1—H1W···F1^ii^	0.86	1.88	2.7014 (16)	161
O1—H2W···F4	0.86	1.94	2.7927 (16)	172

Symmetry codes: (ii) −*x*+3/2, *y*+1/2, −*z*+1/2.
